# Dystrophic Cardiomyopathy—Potential Role of Calcium in Pathogenesis, Treatment and Novel Therapies

**DOI:** 10.3390/genes8040108

**Published:** 2017-03-24

**Authors:** Victoria P. A. Johnstone, Helena M. Viola, Livia C. Hool

**Affiliations:** 1 School of Human Sciences, The University of Western Australia, Crawley, WA 6009, Australia; vicky.johnstone@uwa.edu.au (V.P.A.J.); helena.viola@uwa.edu.au (H.M.V.); 2 Victor Chang Cardiac Research Institute, Sydney, NSW 2010, Australia

**Keywords:** duchenne muscular dystrophy, L-type calcium channel, mitochondria, calcium, cytoskeleton, molecular therapeutics, cardiomyopathy

## Abstract

Duchenne muscular dystrophy (DMD) is caused by defects in the *DMD* gene and results in progressive wasting of skeletal and cardiac muscle due to an absence of functional dystrophin. Cardiomyopathy is prominent in DMD patients, and contributes significantly to mortality. This is particularly true following respiratory interventions that reduce death rate and increase ambulation and consequently cardiac load. Cardiomyopathy shows an increasing prevalence with age and disease progression, and over 95% of patients exhibit dilated cardiomyopathy by the time they reach adulthood. Development of the myopathy is complex, and elevations in intracellular calcium, functional muscle ischemia, and mitochondrial dysfunction characterise the pathophysiology. Current therapies are limited to treating symptoms of the disease and there is therefore an urgent need to treat the underlying genetic defect. Several novel therapies are outlined here, and the unprecedented success of phosphorodiamidate morpholino oligomers (PMOs) in preclinical and clinical studies is overviewed.

## 1. Introduction

Duchenne muscular dystrophy (DMD) is a fatal, X-linked recessive muscle wasting disease that affects 1:3500 of live male births [1]. DMD occurs as a result of inherited or spontaneous out-of-frame mutations in the *DMD* gene that lead to premature termination of translation and a complete loss of dystrophin protein in muscle cells. Dystrophin is a key regulator of mechanical stability within cells, providing a vital link between the sarcomeric cytoskeleton and the extracellular matrix via a complex of transmembrane proteins (dystrophin associated protein complex) [2]. Loss of dystrophin leads to instability of the plasma membrane, inefficient shunting of intracellular contractile forces to the extracellular matrix, and a resultant progressive weakening of striated muscle [3]. Affected patients tend to display early signs of motor weakness between ages three and five and lose ambulation by age 12 [4]. Although cardiomyopathy is ubiquitous in the majority of DMD patients, it has been historically underdiagnosed due to physical inactivity of patients and respiratory complications that obscure clinical detection. Increased survival of patients to more advanced ages has led to the emergence of cardiomyopathy as a leading cause of death from DMD [5]. Understanding the pathogenesis of cardiomyopathy associated with the disease, is crucial to the development of cardioprotective therapies.

## 2. Cardiomyopathy Associated with Duchenne Muscular Dystrophy

### 2.1. Overview

Approximately 95% of patients with DMD develop cardiomyopathy by 20 years of age, and, of these, 20% die from cardiac complications [6]. Mortality associated with DMD cardiomyopathy is becoming increasingly prominent with the advent of interventions, such as assisted ventilation and corticosteroid treatment that prolong life [7]. Cardiomyopathy presents in the early stages of the disease as abnormalities in the electrocardiogram and sinus tachycardia [5]. By adulthood, cardiovascular magnetic resonance (CMR) reveals fibrosis of the left ventricle and ventricular dilation [8,9]. This is accompanied by rhythm abnormalities that include atrial flutter, sinus arrhythmia and frequent premature atrial and ventricular beats [10]. Ventricular arrhythmias are prevalent in patients with impaired ventricular function and are thought to be indicative of progressive myocardial decline [11,12].

### 2.2. Cellular Pathology of Cardiac Dystrophy

The importance of dystrophin in providing cell stability during contraction is well understood (for review see [3,13,14,15]). It acts as an anchor, connecting with laminin α2 (merosin) at the C-terminus through the dystroglycan complex, and cytoskeletal actin at the N-terminus and spectrin-like repeats 11–17 in the rod domain [16]. Absence of dystrophin renders both skeletal and cardiac muscle cells more susceptible to damage upon contraction [17,18,19]. There is good evidence to suggest that excess intracellular calcium is a key trigger of cell death and fibrosis [19], and we have shown that this is in part due to augmented flux via the L-type calcium channel [20] (see Section 4.3 for review). In skeletal muscle, downstream consequences of augmented intracellular calcium include over activation of calcium-dependent proteases, release of caspases and activation of mitochondrial damage pathways, all of which may culminate in apoptotic or necrotic cell death [see 6 for review]). Altered inflammation, impaired vascular adaptation and fibrosis are likely to be key secondary events in the dystrophic patho-cascade [19].

#### 2.2.1. Elevated Intracellular Calcium

##### Mechanical Damage and Membrane Tears

Patients with DMD have historically been categorised as having excessively fragile muscle fibres [6,21,22]. Dystrophin and dystrophin-associated proteins (and accessory proteins, e.g., Vinculin, desmin and spectrin) normally form rib-like lattices known as costameres on the cytoplasmic face of the sarcolemma. Costameres act as mechanical couplers to distribute forces generated in the sarcomere laterally through the sarcolemma to the basal lamina [23]. An early theory was that absence of dystrophin in skeletal muscle and consequent disruption of the costameric lattice rendered the membrane fragile. Indeed, one of the hallmarks of DMD is an elevation of plasma creatine kinase, suggesting that there is increased permeability of the plasma membrane allowing soluble muscle enzymes to leak out of the cell. Increases in membrane permeability have been repeatedly confirmed in a mouse model of DMD (the *mdx* mouse), in studies in which markers that are normally membrane impermeant (such as albumin and Evans Blue dye) are taken up into skeletal muscle fibres [24,25]. The mechanisms that lead to increased permeability of the plasma membrane are poorly understood, but it is widely acknowledged that augmentation of calcium influx contributes to the molecular progression of the disease. A commonly touted theory is that muscle contraction in the absence of dystrophin causes mechanical injury (membrane tears). Indeed, employment of membrane sealants to counteract membrane tears has been shown to restore near normal levels of calcium flux, and improved left ventricular end diastolic volume in *mdx* mice [26,27]. Although membrane tears are likely to play a role in the pathophysiology of DMD, other mechanisms underlying increased membrane permeability (including altered channel activity and increases in reactive oxygen species; ROS) may also be key contributors (reviewed by [28]).

##### Stretch-Activated Channels (SACs) and Mechano-Sensitive Transient Receptor Potential (TRP) Channels

There is some evidence to suggest that stretch-activated channels (SACs) play a role in augmenting calcium influx in muscle fibres [29]. SACs are opened following stretched muscle contraction and allow the non-selective flux of cations [30]. SACs have been shown to be more active in the skeletal muscle of *mdx* mice [31] and in humans with DMD [32]. Inhibitors of SACs such as Gd^3+^, streptomycin and GsMTx-4 inhibit stretch-induced increases in intracellular calcium in *mdx* cardiomyocytes [27,33]. GsMTx-4 has shown particular promise in pre-clinical studies [27,33], and has been granted Orphan drug designation by the U.S. FDA (AT-300, Akashirx, Cambridge, MA, USA). The subunit composition of SACs is currently not known, and it is likely that there are several types of SACs expressed in cardiac muscle. SACs composed of mechano-sensitive transient receptor potential cation (TRPC) channels are thought to be critical in dystrophic cascades, with TRPC1, TRPC3, TRPC6 and TRPV2 as potential candidates. TRPC1 channels are activated by the ROS-dependent Src kinase, leading to an influx of calcium—and the expression levels of this channel increase with age in the *mdx* mouse heart [30]. Evidence for a role of TRPC3 is mixed. TRPC3 knock-out mice display a dystrophic phenotype [34], but stretch-induced contraction of TRPC3 −/− cardiomyocytes does not induce an increase in intracellular calcium [35]. Inhibition of TRPC6 channels in the *mdx* heart alters contractility and arrhythmias [35], and TRPV2 expression is increased in isolated *mdx* cardiac myocytes [36] when compared with wild-type. In addition, inhibition of TRPV2 channels with non-specific blockers and targeted siRNAs reduces influx of calcium upon osmotic stress [36].

##### Store-Operated Calcium Release

A role for calcium stores in mediating increases in intracellular calcium in dystrophin-deficient muscle remains controversial. There are three routes by which calcium gains entry into and out of the sarcoplasmic reticulum (SR) in the heart. The sarco/endoplasmic reticulum Ca^2+^ ATPase (SERCA) is responsible for pumping calcium back into the SR when cytosolic calcium levels increase [37], and there is good evidence to show that function of SERCA is compromised in DMD. Indeed, increasing SERCA expression within dystrophic muscles in transgenic mice, or via viral-mediated delivery, restores calcium homeostasis and slows the disease progression, at least in skeletal muscles [38,39]. Whether expression levels remain unchanged in the *mdx* heart [40] or are down-regulated [41] is unclear, perhaps as a result of different methods used to analyse expression. 

Ryanodine receptors (RyRs) regulate store release of calcium and participate in calcium-induced-calcium-release, an important process for excitation-contraction coupling in the heart. It is possible that RyRs are hyper-phosphorylated by either protein kinase A (PKA) [42] or calcium/calmodulin-dependent kinase II (CaMKII) [42,43,44], rendering them more sensitive to calcium [13]. Downstream effects of hyperphosphorylation of RyRs include dissociation from calstabin-1 (a stabilising protein) [45], disruption of calcium homeostasis and consequent leakiness of the SR, ultimately leading to heart failure [46]. Preventing depletion of calstabin-1 in cardiac muscle using S107, a compound that enhances the binding affinity of calstabin-1 to the RyR, has been shown to inhibit SR calcium leak, and prevent arrhythmias in vivo [47].

Inositol trisphosphate (IP_3_) receptors are a second calcium release channel on the SR activated by IP_3_, a downstream product of phospholipase C (PLC). Early studies in skeletal muscle showed that IP_3_ levels were increased two- to three-fold in dystrophic human and mouse cells lines [48], and application of PLC inhibitors have since been shown to significantly reduce intracellular calcium levels in isolated *mdx* cardiomyocytes when compared to myocytes from wild type (*wt*) hearts [49].

#### 2.2.2. Downstream Consequences of Elevated Intracellular Calcium in the Dystrophic Heart

Whilst the mechanisms leading to increased calcium in the dystrophic heart are still the subject of investigation, it is clear that raised intracellular calcium correlates with the pathology cascade. How does raised resting intracellular calcium lead to heart failure? It is likely that over activation of calcium-dependent cascades occurs in dystrophic muscle, ultimately leading to cell death and/or necrosis [33]. One possibility is that calcium-activated calpains degrade troponin I, leading to contractile dysfunction due to decreased maximal calcium-activated force and decreased calcium sensitivity (discussed in [33]). Metabolic alterations and calcium redistribution into organelles such as the mitochondria and SR may also be involved [33,50]. Overall, it is clear that as the disease progresses muscle repair cannot sufficiently compensate for damage, and replacement of cardiomyocytes with fibrotic tissue occurs [51]. The net result is impaired contractility leading to progressively more critical stages of heart failure.

#### 2.2.3. Functional Muscle Ischemia

One of the earliest histological changes observed in dystrophic muscle is the appearance of small groups of muscle fibres at the same stage of necrosis or regeneration, surrounded by histologically normal muscle fibres [52]. Prior to the discovery of dystrophin, this was hypothesised to result from local microvascular ischemia, and could be replicated in the skeletal muscles of healthy animals by occlusion of intramuscular arterioles with dextran beads or via arterial ligation and vasoconstrictor injection [53,54,55]. It is now clear that functional muscle ischemia mediated by loss of sarcolemmal neuronal nitric oxide synthase µ (nNOSµ) plays an important role in DMD pathology in both skeletal and cardiac muscle [56,57,58]. nNOSµ is a dystrophin-associated protein that is a muscle-specific splice variant of NOS. The nNOSµ is indirectly coupled to dystrophin via α-syntrophin [59,60,61,62], and attenuates vasotone in the working muscle via production of NO [63,64,65,66,67]. Dystrophin deficiency causes displacement of nNOSµ from the sarcolemma to the cytosol, leading to a dysregulation of vasotone and unopposed vasoconstriction [63,66,68]. Transgenic upregulation of nNOSµ in the *mdx* mouse decreased inflammatory markers in the heart and improved stroke volume and ejection fraction [69]. NOS-dependent repeated bouts of functional ischemia coupled with dystrophic muscle fibres that are already weakened is proposed to further promote use-dependent focal muscle injury [70].

#### 2.2.4. Cardiac Mitochondrial Dysfunction

In addition to regulating energy metabolism, mitochondria play critical roles in maintaining calcium homeostasis, producing ROS and mediating cell death via apoptosis and necrosis [71,72,73]. There is mounting evidence to suggest that mitochondrial dysfunction plays a central role in the pathogenesis of DMD-associated heart failure, and altered mitochondrial energy production is indeed one of the first pathophysiological changes to occur in the *mdx* heart, prior to the onset of any detectable cardiomyopathy [74]. Hearts from 10 to 12 week old *mdx* mice demonstrate altered substrate metabolism, including a shift from long chain fatty acids (LCFAs) to carbohydrate (CHO) oxidation for energy production, which is associated with a 20% reduction in activity of the citric acid cycle enzyme aconitase [75]. In addition to marked changes in metabolism, increased intracellular calcium is purported to initiate cascades that trigger necrosis of the mitochondria via a downstream change in mitochondrial membrane permeability. The mitochondrial permeability transition pore (PTP) exhibits excessive opening during stress conditions in *mdx* hearts when compared to *wt* hearts [76], and there is evidence in skeletal muscle suggesting that this may lead to swelling and ultimately rupture of the mitochondria [77].

Our work has shown that loss of dystrophin in *mdx* hearts leads to impaired communication between the L-type calcium channel (LTCC) and the mitochondria [20,78]. The LTCC regulates influx of calcium that is required for excitation and contraction, but also regulates mitochondrial function via transmission of movement of the auxiliary beta subunit through cytoskeletal proteins [79,80,81,82]. In the *mdx* mouse, activation of the LTCC in isolated cardiomyocytes fails to induce increases in mitochondrial membrane potential and metabolic activity observed in wild type animals [20,78]. The implications of this will be discussed in further detail in Section 4.

## 3. Overview of Therapy for DMD-Associated Cardiomyopathy

### 3.1. Current Therapies

Historically, patients with DMD died from pulmonary complications with only a small fraction suffering from terminal cardiomyopathy. Advances in respiratory care that include assisted ventilation, prevention and management of respiratory tract infections with vaccines and antibiotics, together with other auxiliary therapies have resulted in an increase in life expectancy from approximately 14 years in 1960 [83] to 30 or 40 years of age, according to recent studies [84,85]. This prolonging of life has unveiled a new set of clinical challenges, as an increasing number of patients now suffer from a dilated cardiomyopathy that is ultimately fatal [86]. There is currently no cure for cardiorespiratory failure associated with DMD, and treatment strategies are limited to management of symptoms. Existing management guidelines recommend an echocardiogram at six years of age, and then at 1–2 year intervals with annual echocardiograms from age 10 onwards [87]. Cardiac magnetic resonance (CMR) imaging is also emerging as a feasible method for early detection of cardiac pathology [87].

#### 3.1.1. Corticosteroids

Daily corticosteroid therapy is considered the “gold-standard” for DMD treatment [87,88], and its role in improving strength and function of both skeletal and cardiac muscles has been well documented [89,90]. Corticosteroids have anti-inflammatory properties [84,85], and their benefits may include inhibition of muscle proteolysis [91,92], stimulation of myoblast proliferation [93], stabilisation of muscle fibre membranes [94], increase in myogenic repair [95], reduction of cytosolic calcium [96,97] and upregulation of utrophin [98]. Importantly, children treated with corticosteroids prior to the onset of cardiomyopathy show slower progression of heart disease, with an estimated 4% delay in the onset of cardiomyopathy for each year of steroid treatment [99]. This suggests that there may be a critical therapeutic window for the use of steroid therapy in DMD-cardiomyopathy. Whilst the efficacy of corticosteroids as a treatment for DMD has been well-established, undesirable side effects associated with their use render them unsatisfactory as a long-term therapy [13,100], and they remain a supportive therapy only.

#### 3.1.2. ACE Inhibitors and Beta Blockers

Angiotensin-converting enzyme (ACE) inhibitors are used as a first-line treatment in all DMD patients with reduced left ventricular (LV) systolic function, independent of clinical symptoms. ACE inhibitors block the conversion of angiotensin-I to angiotensin-II, thereby limiting the induction of genes that promote cardiac remodelling [101]. ACE inhibitors also decrease peripheral vascular resistance, improve endothelial function, prevent myocardial fibrosis and reduce afterload, all of which delay the progression of heart failure [102,103]. Beta blockers reduce sympathetic input to the heart, and are beneficial in arrhythmic patients [13]. Clinicians frequently prescribe a cocktail of ACE inhibitors, beta blockers and mineralocorticoid receptor antagonists to improve LV systolic function and retard progressive cardiac dysfunction in DMD patients [104,105]. It is becoming increasingly apparent that early treatment prior to the onset of cardiomyopathy is essential, particularly as a large fraction of DMD patients are asymptomatic as a result of limited physical activity, despite suffering advanced cardiomyopathy [106].

### 3.2. Future Therapies

#### 3.2.1. Membrane Repair

Membrane tears are thought to play a role in DMD pathophysiology, and agents such as Poloxamer 188 (P188; poly(ethylene oxide)_80_-poly(propylene oxide)_27_-poly(ethylene oxide)_80_) and mitsugumin 53 (MG53) have been proposed as reparative agents. P188 inserts into artificial lipid monolayers and is capable of repairing damaged biological membranes [107]. Studies in *mdx* heart have shown that administration of P188 during a dobutamine-mediated stress protocol prevents the development of heart failure [26]. In a canine model of DMD (golden retriever muscular dystrophy; GRMD), chronic infusion of P188 resulted in significantly decreased cardiac fibrosis and prevention of dilated cardiomyopathy [108]. MG53 is an essential component of the membrane repair cascade in striated muscle, and injection of recombinant MG53 prevents exercise-induced skeletal muscle damage in *mdx* mice [109] Compounds that promote membrane repair may be of particular importance in offering immediate benefit during times of increased stress, such as respiratory failure and decompensated heart failure [86].

#### 3.2.2. Utrophin Up-Regulation

Utrophin is a homologue of dystrophin that exhibits limited expression in the neuromuscular and myotendinous junctions in normal muscle [110], and is thought to be an important regulator of actin filament length during neuromuscular development [111]. In *mdx* mice and DMD patients, utrophin is overexpressed throughout the sarcolemma of muscle fibres, presumably as a surrogate protein to compensate for the lack of dystrophin. Despite functional differences between utrophin and dystrophin, the two proteins share many of the same binding partners and as such upregulation of utrophin is considered a potentially viable therapy to minimise dystrophic degeneration [100]. Many pharmacologic agents have been evaluated in pre-clinical studies for upregulation of utrophin, including nabumetone [112], heregulin [113], _L_-arginine [114], a peroxisome proliferator-activated receptor agonist (GW501516) [115], trans-activator of transcription (TAT)-utrophin [116], RhoA [117] and recombinant biglycan (rhBGN) [118]. Each has been shown to be capable of improving dystrophic muscle pathology to varying degrees. In addition, SMT C1100 is a particularly promising compound that induces utrophin upregulation, is currently entering Phase 2 studies and has recently been granted Fast Track designation by the FDA. 

#### 3.2.3. Stop-Codon Read-Through Therapy

Approximately 10%–15% of patients have a single base change in the DMD gene that leads to a premature stop-codon, thereby disrupting the open reading frame [119,120]. A potential treatment strategy with some promise involves suppression of the stop codon mutation, a method that has shown efficacy in DMD patients. In one study, treatment of DMD patients with the aminoglycoside antibiotic gentamicin for 6 months was reported to cause up to a 15% increase in skeletal muscle dystrophin levels [121]. In these patients muscle strength was normalised and a modest increase in forced vital capacity was reported. It was concluded that larger doses would have to be administered to induce clinically relevant effects, and concerns over toxicity and long-term intravenous administration led to the development of translarna (originally named ataluren), an orally administered equivalent [122]. Translarna was trialled in a Phase 3 randomised, double-blind, placebo-controlled study in DMD patients where the drug was well tolerated without any signs of off-target effects [123]. Functional outcomes from the study were disappointing overall however, with consistent improvement in ambulation reported only in some subgroups. In addition, there was no mention of favourable cardiac outcomes at the conclusion of the study [123,124,125]. Translarna is currently licensed for European use, although is likely to be withdrawn in Germany. Despite largely disappointing results, a New Drug Application is currently under review for Translarna by the FDA with outcomes expected to be announced in October 2017.

#### 3.2.4. Viral Gene Therapy

Another strategy involves the use of viral vectors to deliver a dystrophin transgene to the cell. A major constraint of AAV gene therapy is the limited packaging capacity of the virus capsid, thereby preventing delivery of the full dystrophin cDNA. This has led to the insertion of shorter truncated versions instead (mini- or micro-dystrophin) [126], that have yet to be functionally tested in humans [100]. The first cardiac gene therapy study was performed in neonatal *mdx* mice using an AAV-5 microgene vector, which was shown to restore dystrophin and other dystrophin-associated proteins, and increase the strength of the cardiomyocyte membrane [127]. Further research using the same microgene demonstrated positive cardiac dystrophin expression, normalisation of electrocardiographic (ECG) defects and improvement of cardiac hemodynamics when administered in both young and adult *mdx* mice [128,129,130,131]. An AAV-9 microgene vector has subsequently been shown to induce efficient whole-heart gene transfer, even in the presence of extensive myocardial fibrosis [132,133]. In near terminal-age mice (20 months old), fibrosis was significantly reduced and hemodynamic performance significantly enhanced [133], but the same could not be achieved in terminal-age mice (greater than 21 months old) [132]. Despite these positive results, there are major obstacles for gene therapy that must be overcome before evaluation in DMD boys. Firstly, immunological responses to the virus have been observed in larger animals and humans [134,135,136], and this is of particular concern since the human population carries antibodies towards prevalent viruses such as AAV [120,137]. An immune response from dystrophin is also a concern, as patients will not have been exposed to some (or all) of the epitopes of dystrophin [138]. Immunosuppression may be implemented to reduce these risks [137], but carries with it another set of problems. In addition, repeat administration is likely to be problematic due to the presence of neutralising antibodies as a consequence of the first administration [139].

#### 3.2.5. Cell-Based Therapy

Transplantation of healthy dystrophin-expressing cells into DMD patient tissue is a relatively promising therapy, and to date several different muscle precursor cells have been tested in preclinical studies, including myoblasts, fibroblasts, bone-marrow derived stem cells, CD133+ stem cells, mesangioblasts and iPS cells [140,141,142,143,144,145,146,147,148]. As with gene therapy, immune responses are a major concern with a cell-based therapeutic approach [149]. To circumvent this, several groups have utilised genetically modified patient precursor cells as a treatment. The genome of the host cells is modified in vitro using transcription activator-like effector nucleases (TALENs) and clustered regularly interspaced short palindromic repeats (CRISPR)-cas9 for splicing correction of the dystrophin gene to induce exon skipping, correct the mutation, shift the reading frame or insert the missing exon [150,151,152]. It is worth noting that CRISPR-cas9 has also been delivered using an AAV approach [153]. Such approaches have proven effective in some instances [151], but the ability to induce and sustain adequate dystrophin levels has been a challenge. Additionally, cell delivery is currently most successful if performed intramuscularly [120], but this allows treatment of only one muscle at a time and would exclude treatment of the diaphragm and heart. In addition, high titres of modified cells are required to significantly reverse the dystrophic phenotype.

Human trials using transplanted cells have yielded some promising results [154], but insufficient numbers and distribution of transplanted cells has been a problem [155]. Ongoing trials are working to optimise transplant procedures [156], but ultimately a means of delivering to cardiac and respiratory muscle will be essential before this technique is a viable therapy in humans.

#### 3.2.6. Endocrine Mediators

Some success has been reported using endocrine mediators and their targets for gene therapy. For example, treatment with the corticoid releasing factor (CRF) receptor agonist urocortin has had beneficial effects on skeletal muscle structure and function in *mdx* mice [157]. Regulation of the myostatin pathway is another area of current investigation. Myostatin is a physiological antagonist of insulin-like growth factor-1 (IGF01), and mediates muscle atrophy. Blocking myostatin has proven successful in pre-clinical studies [158] and a Phase 2 multicentre trial is currently underway (DMD Myostatin Trial).

#### 3.2.7. Targeting Signalling Pathways

Pre-clinical studies have also focused on targeting the multiple aberrant signalling pathways that are linked with the pathogenesis of DMD. Antioxidant therapy delays the onset of dilated cardiomyopathy and improves lifespan in *mdx* mice, perhaps as a result of reduced telomere erosion [159]. There is evidence to suggest that oxidative stress activates pro-inflammatory pathways mediated by NF-_Κ_B in skeletal muscle [160], and indeed blunting of the NF-_Κ_B pathway improves cardiac contractility in utrophin/dystrophin deficient double knockout mice [161]. Targeting pro-fibrotic pathways with dietary flavanols reduces cardiac expression of transforming growth factor β1 (TGF-β1) in *mdx* mice, and this was associated with reduced cardiac damage (assessed histologically) [162]. The family of heat shock proteins (Hsps) are induced by cellular stress, and thought to play a role in cellular protection. Various Hsps are upregulated in dystrophic heart [163] and treatment with Hsp-inducers improves membrane integrity, systolic function and reduces collagen deposition in the heart [164].

#### 3.2.8. Antisense Oligonucleotides

##### Overview

Antisense oligonucleotides (AOs) are short, single stranded DNA sequences that may be used to alter exon or splice site selection in order to restore the correct dystrophin reading frame [165]. AOs used for splice modification are complementary in sequence to a target pre-mRNA splice site, and usually target either a specific 5′ or 3′ splice site, or alternatively bind to a splicing regulatory element such as an intronic or exonic splicing enhancer or intronic or exonic splicing silencer [166]. This induces the production of a truncated, but mostly functional dystrophin protein. Exon skipping therapy has the potential to treat 79% of patients with deletion mutations, 91% with small-scale mutations and 73% with duplication mutations [167]. Although mutations are spread across the 79 *DMD* exons, there are specific “hotspot” regions where deletions are common, such as between exons 45 and 55, where approximately 70% of DMD-causing deletions are located [168]. AO sequences have been designed for every internal DMD exon [169], but the majority of therapeutic development has thus far concentrated on skipping those exons that will benefit the greatest number of patients. For example, skipping of exon 51 could potentially benefit 13% of all DMD mutations, exon 45 could benefit 8.1% and exon 53 could benefit 7.7% [167]. Some DMD mutations will require at least 2 exons to be skipped in order to restore or maintain the reading frame, and so-called multiple exon skipping is currently under investigation [170]. In order for an AO to correct splicing defects, it must satisfy several requirements [171]. Firstly, it must not activate RNase H, a ubiquitous enzyme that degrades the RNA strand of an RNA/DNA complex. It must also access the target pre-mRNAs within the nucleus in order to efficiently compete with splicing factors. Several types of modified AOs have been developed that meet these requirements, including AOs with modifications to the 2′ position, such as 2′O-methylated phosphorothioate (2′OMePS), and AOs with backbones based on morpholino structural type (phosphorodiamidate morpholino oligomers; PMOs). Both of these compounds are RNase H inactive, display high nuclease resistance and exhibit a high affinity for target sequences [171]. Both of these compounds have also been evaluated clinically for exon 51 skipping in DMD patients, and are described in further detail below.

##### 2′O-methyl Phosphorothioate AOs

The phosphorothioate backbone contains internucleotide linkages that confer a negative charge, and this facilitates enhanced binding to circulatory proteins and increases the half-life in plasma [172]. Pilot data produced in the early 2000s demonstrated that treatment of cultured *mdx* mouse myoblasts with a 2′O methyl phosphorothioate (2′OMePS) antisense oligonucleotide was capable of inducing exon 23 skipping and restoring dystrophin expression (the *mdx* mouse contains a naturally occurring nonsense mutation in exon 23 of the *DMD* gene) [173,174]. Subsequent work showed that intravenous administration of the same compound in *mdx* mice restored dystrophin expression in skeletal muscles, but not in heart [175]. In 2009, the company developing the 2′OMePS chemistry for commercial exon skipping (Prosensa) partnered with GlaxoSmithKline to bring drisapersen (also known as PRO051 or GSK2402968) to clinical trial. In phase 1–2a of the trial 12 DMD patients were administered drisapersen for five weeks at doses ranging from 0.5 mg/kg to 6 mg/kg, and this was followed by a 12-week extension study with all patients receiving 6 mg/kg [176]. After 25 weeks of continuous treatment, the mean distance covered in the six-minute walk test (6MWT) increased by 31.5 m from baseline, but, by Week 49, the difference between treated and placebo cohorts was no longer significant [177]. Drisapersen was further tested in a phase 3 double-blinded placebo-controlled study (DMD114044) involving 186 boys who were randomised to either drisapersen treatment, administered at a dose of 6 mg/kg/week (*n* = 125) or placebo (*n* = 61) via subcutaneous injection over 48 weeks [178]. There was no statistical significance between drisapersen and placebo groups in ambulation, as assessed via the 6MWT. There was also no difference in key secondary assessments of motor function: the 10-m walk/run test, four-stair climb and North Star Ambulatory Assessment. In addition, many significant treatment-related adverse events were reported, including flu-like symptoms, persistent injection site reactions, kidney inflammatory responses and proteinuria present in 46% of patients [176,177,179]. Many of these adverse events are likely to be a direct result of the phosphorothioate backbone that although confers increased half-life, also results in off-target effects due to its interaction with numerous proteins. For example, phosphorothioate AOs bind to and modulate immune cell receptors such as toll-like receptors (TLRs) [180,181]. This initiates inflammatory cascades that result in elevated expression of cytokines and chemokines as well as irreversible kidney inflammation and loss of function [182]. The disappointing outcomes of the drisapersen trials ultimately resulted in the termination of the GlaxoSmithKline and Prosensa partnership in early 2014, a major blow to the Duchenne community [183].

##### Phosphorodiamidate Morpholino Oligomers

Phosphorodiamidate morpholino oligomers (PMOs) have a six-membered morpholine ring moiety, with phosphorodiamidate linkages joining the rings. They are nuclease and RNase H resistant and, in contrast to phosphorothioates the backbone carries no charge at physiological pH [166]. This reduces the chances of off-target effects from these compounds, and they have indeed been proven non-toxic and very stable, with the majority excreted virtually unchanged via the urine [184]. Preclinical assessment of PMOs has been very successful. PMOs have been used to induce exon skipping [185], and intravenous administration into *mdx* mice elicits widespread dystrophin expression in skeletal muscle [186,187] as well as improving muscle pathology and locomotor activity [188]. Sarepta Therapeutics has developed eteplirsen (AVI-4658), a 30 nucleobase PMO for the treatment of DMD patients with exon 51 skippable deletions that is so far showing unprecedented clinical benefits. In an early proof-of-concept study, intramuscular injections of 0.9 mg of eteplirsen induced exon skipping and positive dystrophin expression in five DMD patients [189]. A subsequent dose escalation study was carried out, and 19 patients were treated with intravenous doses of eteplirsen ranging from 0.5 to 20.0 mg/kg. In this study, all doses induced exon skipping, and positive dystrophin expression was detected in boys dosed with 2 mg/kg and above [190]. Eteplirsen was then trialled in a Phase 2, 24-week randomised placebo-controlled study, in which three groups of four patients (aged 7–13 years) were treated weekly with intravenous eteplirsen at 50 mg/kg, 30 mg/kg or placebo (Study 201). At the conclusion of the study, a significant increase in production of dystrophin in treated patients was achieved, but with no significant difference in 6MWT scores (however, several of the boys in the study were at a young enough age to still be gaining motor function). After 24 weeks, the four patients originally randomised to the placebo group were rolled over to open-label eteplirsen of 30 mg/kg (*n* = 2) or 50 mg/kg (*n* = 2) as a result of the unprecedented benefits reported. All 12 patients then continued receiving weekly eteplirsen in an ongoing extension study (Study 202) (Phase 2b) for over three years [191]. At Week 168, the continuously treated ambulatory patients continued to walk within 18% of their Week 12 distance, while the placebo/delayed treatment cohort continued to walk within 23% of their Week 36 distance, a significant improvement on the rapid loss of ambulation that occurs in DMD patients on standard steroid therapy [192]. No clinically significant treatment-related adverse events have been observed in over more than three years in patients treated with eteplirsen at either 30 mg/kg or 50 mg/kg. There have been no symptoms of immune activation and no report of flu-like symptoms. In addition, pulmonary function has remained stable in these patients, and the decline in respiration that is a hallmark of the disease appears to have been staved off. However, cardiac abnormalities consistent with the underlying disease remain in these patients, and there is therefore an urgent need to identify therapies that restore functional dystrophin expression within cardiac muscle, as well as skeletal and respiratory muscle.

## 4. Reversal of DMD-Associated Cardiomyopathy Following Treatment with Antisense Oligomers

### 4.1. Overview

Dilated cardiomyopathy is a characteristic feature of DMD and is a major cause of morbidity and mortality [193]. Current treatment strategies, including antisense oligonucleotides, have elicited recovery in skeletal and pulmonary function, but with limited ability to prevent progressive cardiac decline. Those in the exon skipping field are now paying particular attention to conjugating or complexing AOs with carrier molecules that might facilitate their uptake into various tissues, including the heart. A large amount of recent work has focused on cell-penetrating peptides (CPPs), short cationic or amphipathic peptides that are capable of delivering various cargoes across the cell membrane, and can be non-covalently conjugated to charged AOs (via electrostatic interaction) or covalently conjugated to uncharged AOs [194]. A recent next-generation PMO-conjugate called Pip6-PMO induces substantial cardiac dystrophin expression in *mdx* mice, and this prevents the exercise-induced progression of cardiomyopathy that is otherwise observed [195]. Interestingly, our data are consistent with recent results from others indicating that only a small amount of dystrophin is required to reverse dysfunction in *mdx* cardiomyocytes [78,196]. This suggests that marginal improvements in uptake strategies that are currently being trialled may be sufficient to prevent cardiac failure associated with DMD. 

### 4.2. The Cytoskeletion as a Link between the L-Type Calcium Channel (LTCC) and the Mitochondria

It is now apparent that ion channels are not unitary structures, but rather are part of large, multi-unit complexes that comprise the ion channels and their auxiliary subunits as well as cytoskeletal elements, regulatory kinases and phosphatases, trafficking proteins, extracellular matrix proteins and perhaps even other ion channels. Disruption of one component of the complex can have significant influence on channel function and localisation, and therefore cellular excitability and function [197]. We have demonstrated that the LTCC regulates mitochondrial function in the heart, and that this is mediated in part due to a structural and functional association via the cytoskeleton [20,78,79,80,81,198,199,200,201]. Activation of the LTCC either with the channel agonist BayK(−) or depolarisation of the plasma membrane with high potassium solution or by voltage-step using the patch-clamp configuration causes an increase in the mitochondrial membrane potential (Ψ_m_)—the electrochemical potential used to drive conversion of ADP to ATP in the mitochondria in the heart. We have shown that this channel-mediated increase is independent of calcium in the cardiomyocyte; it occurs when the mitochondrial calcium uniporter is blocked with Ru360 and under calcium-free conditions [79]. The LTCC therefore is not only critical for regulating calcium influx into the cardiomyocyte, but also plays a key role in regulating mitochondrial energetics. The LTCC is anchored to F-actin and β-tubulin networks by subsarcolemmal stabilising proteins that also act to regulate channel function [82,202,203]. In addition, the mitochondria are proposed to interact with cytoskeletal proteins via docking sites on the outer mitochondrial membrane [204,205,206]. We have shown that depolymerisation of actin with latrunculin A prevents the channel-mediated increase in Ψ_m_ in the heart [79]. In addition, preventing movement of the beta auxiliary subunit of the LTCC (which is tethered to cytoskeletal proteins) with application of a peptide derived against the alpha-interacting domain attenuates the increase in Ψ_m_ [79,198,207]. We have additionally shown that animal models with disordered cytoskeletal networks exhibit impaired regulation of mitochondrial function by the LTCC. Mice expressing the human disease causing cardiac troponin I mutation Gly203Ser (*cTNI-G203S*) exhibit marked myofiber disarray [208], impaired LTCC kinetics and a hypermetabolic mitochondrial state that precedes development of hypertrophic cardiomyopathy [200]. Similarly, heterozygous mice expressing the human disease-causing β-myosin heavy chain mutation Arg403Gln (*αMHC^403/+^*) display altered LTCC function and hyper-energetic mitochondria, leading to hypertrophic cardiomyopathy [209].

We have good evidence that the LTCC plays an important role in the pathophysiology of DMD [20,78]. There have been many clinical studies into the efficacy of calcium channel antagonists (such as verapamil, diltiazem, nidedipine and flunarizine) as a treatment for DMD, but a Cochrane study concluded that such treatments have no overall benefit on muscle function [210]. We argue that altered calcium handling in cardiomyocytes is due to loss of dystrophin that disrupts cross-talk between the LTCC and the mitochondria [20,78]. This leads to contractile dysfunction and reduced energy production in the dystrophic heart. We as well as others have shown that these cellular changes precede the onset of clinical detection of the cardiomyopathy, and as such may offer insight into an ideal therapeutic window within which to target treatment [20,78,211,212].

### 4.3. Altered L-Type Calcium Channel Function in the Mdx Mouse

**Figure 1:**
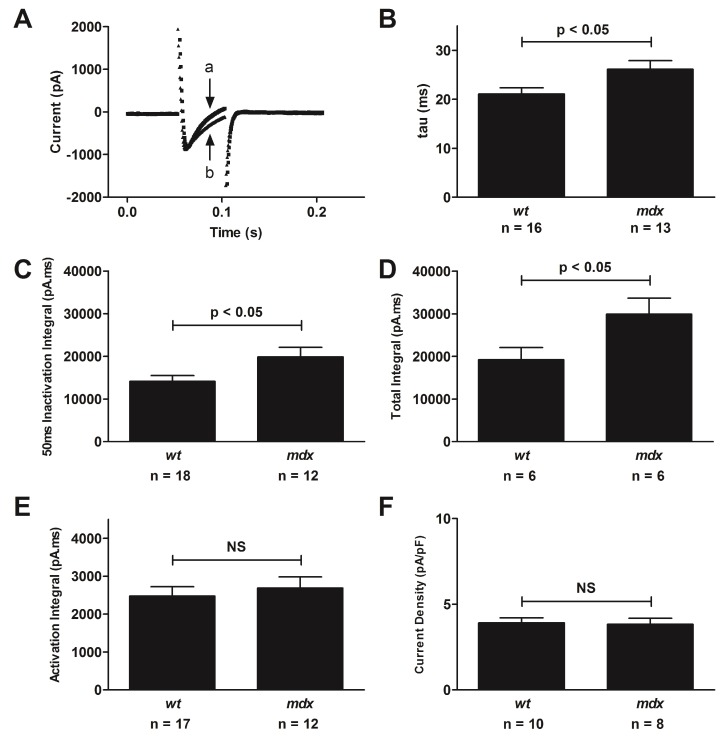
Altered L-type Ca^2+^ channel function in the *mdx* mouse. (**A**) Representative current traces recorded from a wild type (*wt*) [a] (110 pF) and *mdx* [b] (112 pF) myocyte over a 100 ms time course as indicated. Means ± SEM of: (**B**) tau of inactivation; (**C**) 50 ms inactivation integral of current; (**D**) total integral of current; (**E**) activation integral of current; and (**F**) current density for all myocytes as indicated. Reproduced from [20]. NS, non-significant.

The first evidence for impaired function of LTCCs in dystrophic cells came from an early study in which the LTCC blocker nifedipine was shown to inhibit degeneration of *mdx* muscle fibres induced by tetanic stimulation [213]. Subsequent work demonstrated that the kinetics of the channel are altered in the *mdx* model. We monitored LTCC function and biophysical properties in cardiomyocytes isolated from eight-week-old male *mdx* mice using the patch clamp configuration (Figure 1) [20,78]. Consistent with reports from others [211,212,214], we observed a delay in inactivation rate of the channel (Figure 1A,B). In accordance with a delayed inactivation rate, the 50-ms inactivation integral of current and total integral of current were significantly greater in *mdx* myocytes when compared with controls, but the activation integral remained unchanged (Figure 1C–E) [20]. Calcium channel inactivation occurs via a slow, voltage-dependent mechanism and a faster, calcium-independent mechanism [215]. Interestingly, the delayed inactivation persists regardless of whether barium or calcium is used as the charge carrier [211]. This supports the notion that changes in channel inactivation are likely attributed to alterations in cytoskeletal organization due to the lack of dystrophin, rather than due to calcium-dependent processes. Aside from changes in inactivation, at 8 weeks of age other properties of the channel appear normal, with no change in current density (Figure 1F) [20,78,216] and no difference in cell size [20], indicating that channel expression is not altered. Indeed, we have confirmed these findings on immunoblots [78]. In addition, resting membrane potential is the same in *mdx* and *wt* cardiomyocytes [216], suggesting that changes in the refractory period do not underlie the changes in channel kinetics.

### 4.4. Altered Calcium Handling in the Mdx Mouse and Downstream Changes to Mitochondrial Function

**Figure 2:**
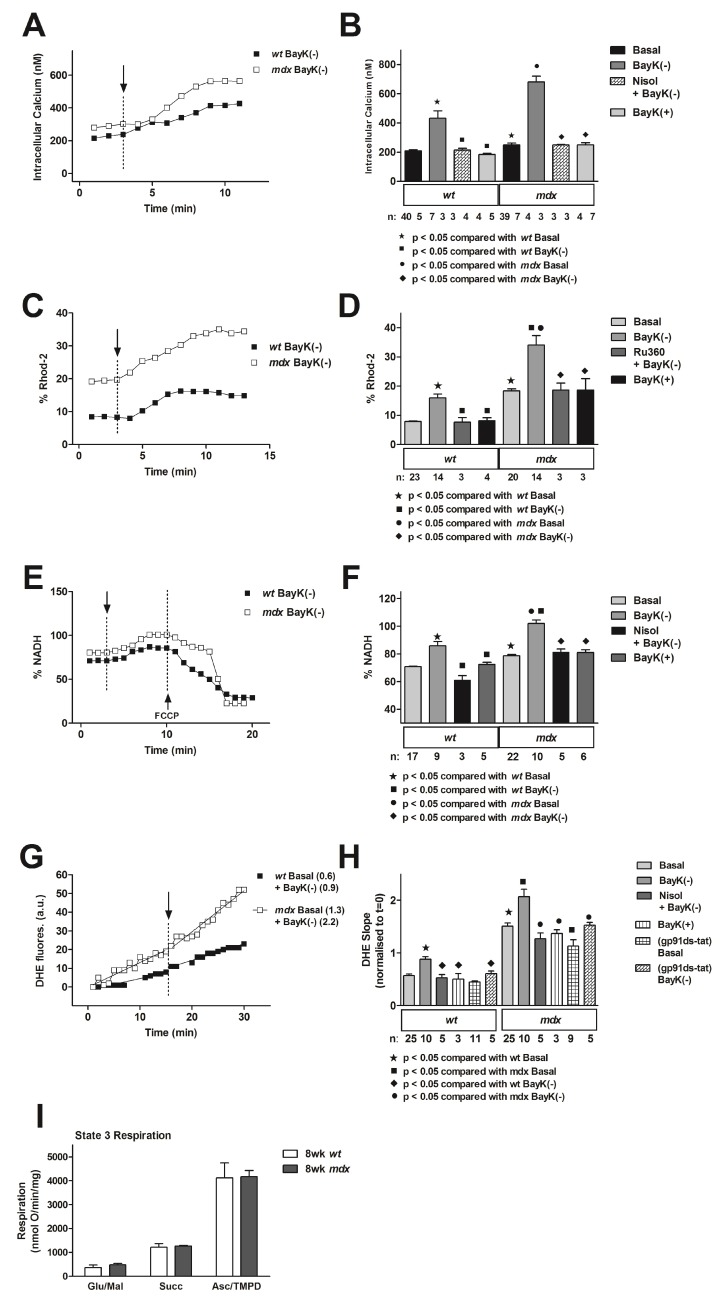
Altered calcium handling and mitochondrial function in the *mdx* mouse. (**A**) Representative traces of intracellular calcium recorded in *wt* and *mdx* myocytes recorded before and after exposure to 10 µM BayK(−) (indicated by arrow); (**B**) Means ± SEM of intracellular Ca^2+^ for all myocytes, exposed to treatments as indicated; (**C**) Representative traces of mitochondrial calcium (recorded as Rhod-2-fluorescence) recorded from *wt* and *mdx* myocytes before and after exposure to 10 µM BayK(−) (indicated by arrow); (**D**) Means ± SEM of changes in Rhod-2-fluorescence after addition of BayK(−) for *wt* and *mdx* myocytes; (**E**) Representative traces of NADH autofluorescence (%NADH) recorded from *wt* and *mdx* myocytes before and after exposure to 10 µM BayK(−) (indicated by arrow). Ten-micromolar FCCP was added as indicated to confirm the signal was mitochondrial in origin. (**F**) Means ± SEM of changes in NADH fluorescence for all myocytes exposed to treatments as indicated. (**G**) Representative traces of dihydroethidium (DHE) fluorescence recorded from *wt* and *mdx* myocytes before and after exposure to 10 µM BayK(−) (indicated by arrow). Slopes of the signals are indicated in brackets at right. (**H**) Means ± SEM of changes in DHE slope for all myocytes exposed to treatments as indicated.; (**I**) Respiration and mitochondrial electron transport chain complex activity in mitochondria isolated from eight-week-old *wt* and *mdx* hearts. Measurements performed in triplicate. n, number of myocytes; Nisol, 10 µM nisoldipine; BayK(+), 10 µM; Ru360, 10 µM; gp91ds-tat peptide, 10 µM; FCCP, 10 µM Carbonyl cyanide-4-(trifluoromethoxy)phenylhydrazone; a.u., arbitrary units. Reproduced from [20].

Changes in calcium homeostasis underlie the development of many cardiomyopathies, and elevations in intracellular calcium and the downstream activation of protein degradation and necrotic pathways are heavily implicated in DMD disease progression [217,218]. Our findings demonstrate that isolated *mdx* mouse myocytes exhibit a greater influx of calcium and significantly raised resting levels of calcium than *wt* controls (Figure 2A,B) [20]. One consequence of elevated resting cytoplasmic calcium is an increase in resting mitochondrial calcium. We assessed changes in Rhod-2 fluorescence in isolated myocytes from eight-week-old *mdx* mice and suggested that resting mitochondrial calcium was elevated, but also demonstrated that flux of calcium into the mitochondria upon activation of the channel was unchanged compared to *wt* controls (Figure 2C,D) [20]. Increases in mitochondrial calcium trigger upregulation of the tricarboxylic acid (TCA) cycle, resulting in increased production of reduced nicotinamide adenine dinucleotide (NADH) from oxidised nicotinamide adenine dinucleotide (NAD^+^). In line with this, we detected channel-activated increases in NADH production (assessed as NADH autofluorescence) (Figure 2E,F). We also monitored superoxide production (assessed as DHE fluorescence). Previous studies have demonstrated increased NAD(P)H-oxidase expression and associated increase in superoxide production in the *mdx* heart [33]. Consistent with this, we found that basal superoxide was increased in *mdx* cardiomyocytes (Figure 2G,H) that was due to increased NAD(P)H-oxidase activity, because application of gp91ds-tat peptide to inhibit its activity decreased *mdx* resting levels of superoxide (Figure 2H). Activation of the LTCC led to further increases in superoxide production, and this was demonstrated to be mitochondrial in origin, as it was blocked with application of mitochondrial uniporter blocker Ru360 and complex III inhibitor myxothiazol, but was unaffected by gp91ds-tat peptide (Figure 2H). In addition, respiration in mitochondria isolated from eight-week-old *mdx* mice was shown to be similar to respiration in *wt* mice, indicating that deficiencies in mitochondrial function only occur in the intact myocyte (Figure 2I). These data suggest that alterations in calcium handling and downstream disturbances in mitochondrial function result from a disrupted cytoskeleton in *mdx* mice, and these perturbations are likely to be critical in the development of dystrophic cardiomyopathy.

### 4.5. Recovery of Regulation of Mitochondrial Function by the L-Type Calcium Channel Following PMO Treatment

**Figure 3:**
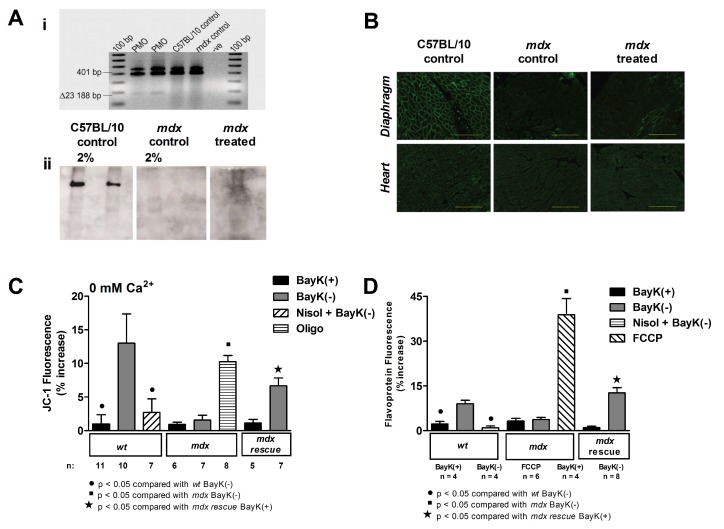
Recovery of functional link between the L-type calcium channel and the mitochondria in *mdx* cardiomyocytes after treatment with a PMO to skip exon 23. (**A**) (i) RT-PCR performed on cardiac muscle RNA from *mdx* mice treated with PMO demonstrating exon 23 skipping (Δ23); and (ii) Immunoblot performed on cardiac muscle from C57BL/10 control mice, untreated *mdx* mice (*mdx* control), and mice treated with PMO, demonstrating presence of dystrophin in control and *mdx* treated panels. Two per cent and one per cent dilutions are shown; (**B**) Immunostaining of heart and diaphragm cryosections from a C57BL/10 control mouse, untreated *mdx* mouse (*mdx* control), and *mdx* mouse treated with PMO demonstrating presence of dystrophin (*mdx* treated). Scale bars: 100 µm; (**C**) Mean ± SEM of increases in JC-1 fluorescence for all *wt*, *mdx* and *mdx* myocytes, including myocytes from *mdx* treated with PMO (“*mdx* rescue”) exposed to treatments as indicated. Oligomycin induced a robust increase in JC-1 signal in *mdx* myocytes; (**D**) Mean ± SEM of increases in flavoprotein fluorescence for all *wt*, *mdx* and *mdx* myocytes, including myocytes from *mdx* treated with PMO (“*mdx* rescue”) exposed to treatments as indicated. FCCP was added to increase flavoprotein signal, confirming the signal was mitochondrial in origin. Nisol, 10 µM nisoldipine; Oligo, 20 µM oligomycin; FCCP, 10 µM FCCP. Reproduced from [79].

We have used exon skipping strategies to induce recovery of regulation of mitochondrial function by the LTCC in *mdx* mice [78]. Mice were treated at 20 mg/kg/wk with a PMO targeting mouse dystrophin exon 23 for a total of 24 weeks. We confirmed that the treatment induced exon skipping in cardiac muscle via RT-PCR, and used immunoblot and immunohistochemistry staining on cryosections to verify that a functional, but truncated, dystrophin protein was expressed (Figure 3A,B). Experiments on isolated cardiomyocytes from treated *mdx* mice revealed that the channel-activated increases in Ψ_m_ and flavoprotein oxidation were partially recovered in these animals (Figure 3C,D) [78]. This suggests that dystrophin is a key component of the structural and functional link between the LTCC and the mitochondria, and that loss of this protein in the *mdx* mouse disrupts normal regulation of mitochondrial function by the channel, resulting in a hypometabolic state within the heart.

## 5. Conclusions

Duchenne muscular dystrophy is underpinned by a complex pathophysiology involving numerous signalling cascades. The extent to which the cellular disease mechanisms differ between skeletal and cardiac muscle remains unknown, but changes in calcium handling and mitochondrial dynamics are critical in establishing the phenotype. Corticosteroids have been successfully used to ameliorate the skeletal muscle phenotype for a limited time, and death from respiratory failure is declining due to the advent of better supportive strategies such as mechanical ventilators and management of respiratory tract infections with antibiotics and cough assistance. The therapeutic focus has therefore shifted towards developing treatment for dystrophic cardiomyopathy. Patients with better motor and pulmonary function are living longer, and engaging in activities that exert a greater cardiac load. Whilst pharmacological interventions such as ACE inhibitors and beta blockers can stabilise cardiac function to some extent, morbidity and mortality from heart failure in DMD patients remains high and there is an urgent need to develop treatments that prevent both motor, pulmonary and cardiac decline. Promising results from the eteplirsen study are at the current forefront of this field, and it is anticipated that minor modifications to the existing chemistry that improve uptake of the compound into the heart may greatly delay death from cardiorespiratory failure in DMD boys with the relevant mutation.
